# Anticoagulants and Dementia: A Systematic Review

**DOI:** 10.7759/cureus.39693

**Published:** 2023-05-30

**Authors:** Amy E Kalloo, Ethan Slouha, Connor P Gallagher, Ziyad Razeq, Vasavi Rakesh Gorantla

**Affiliations:** 1 Clinical Sciences, St. George's University School of Medicine, True Blue, GRD; 2 Anatomical Sciences, St. George's University School of Medicine, True Blue, GRD

**Keywords:** warfarin, vitamin k antagonists, antithrombotics, atrial fibrillation, dementia, anticoagulants

## Abstract

Many patients diagnosed with atrial fibrillation (AF) develop dementia. Most AF patients are also prescribed some antithrombotic medication to reduce the incidence of stroke, as clots can form within the left atrium. Some research has found that, excluding patients who have experienced strokes, anticoagulants may serve as protective agents against dementia in AF. This systematic review aims to analyze the incidence of dementia in patients who were prescribed anticoagulants. A comprehensive literature review was conducted using the databases PubMed, ProQuest, and ScienceDirect. Only experimental studies and meta-analyses were chosen. The search included the keywords "dementia and anticoagulant" and "cognitive decline and anticoagulants". Our initial search generated 53,306 articles, which were narrowed down to 29 by applying strict inclusion and exclusion algorithms. There was a decreased risk of dementia in patients who had been prescribed oral anticoagulants (OACs) in general, but only studies investigating direct oral anticoagulants OACs (DOACs) suggested that they were protective against dementia. Vitamin K antagonist (VKA) anticoagulants showed conflicting results, with some studies indicating they might increase the risk for dementia, while others suggested that they are protective against it. Warfarin, a specific VKA, was mainly shown to reduce the risk of dementia but was not as effective as DOACs or other OACs. Lastly, it was found that antiplatelet therapy may increase the risk of dementia in AF patients.

## Introduction and background

Atrial fibrillation (AF) is a cardiac condition caused by a dysfunction in the SA node leading to an irregularly irregular heart rhythm [1]. AF is linked to multiple comorbidities, including stroke and subsequent development of dementia [1]. AF is associated with different types of dementia, such as vascular dementia and Alzheimer's disease. AF and coexisting cardiovascular disease increases the risk of dementia [1]. Anticoagulants are used in the management of AF to reduce the risk of blood clots and complications such as cerebrovascular accidents/stroke [2].

Direct oral anticoagulants (DOACs) can directly inhibit thrombin, like dabigatran, or they can inhibit factor Xa, such as rivaroxaban, edoxaban, and apixaban [2]. Thrombin catalyzes the conversion of fibrinogen to fibrin as the final step of the coagulation cascade, ultimately reducing the production and growth of thrombi [2]. Vitamin K antagonists (VKAs), such as warfarin, affect clotting factors II, VII, IX, X, and proteins C and S by decreasing their synthesis [3]. Unfortunately, administering VKAs requires careful monitoring of INR levels to prevent the underactivation of coagulation and their sequelae [3]. VKAs may not be effective in preventing dementia in AF patients because vitamin K has protective effects on neural cells [4]. The lack of vitamin K may make it easier for these neural cells to atrophy or undergo apoptosis [4].

The idea is that anticoagulants allow for a more optimal blood flow throughout the vascular system, thereby decreasing dementia risk, but the mechanism behind it is unclear. This paper aims to evaluate the association between the utilization of anticoagulants and the incidence of dementia in patients with AF.

## Review

Methods

Three independent authors conducted the systematic review based on the inclusion and exclusion criteria, as well as the risk of bias assessment. A comprehensive and extensive literature search was performed using ScienceDirect, PubMed, and ProQuest databases covering the period from January 1, 2002, to December 31, 2022. The keywords included "dementia, anticoagulants, atrial fibrillation, comorbidities, cognitive impairment, complications, and anticoagulants".

The electronic search focused on peer-reviewed, experimental articles deemed to be in line with the scope of this paper. Duplicate articles published before 2022 and articles that were not written in English were excluded during the screening process. Once completed, three co-authors independently reviewed the results. Articles found in the investigation were analyzed based on their title, abstract, study type, and full-text accessibility. The initial search from the three databases yielded 56,306 articles. The selected articles were further narrowed down based on keyword specifics and a preview of the abstracts. According to the inclusion and exclusion criteria, 29 articles were found to be within the scope of interest.

Inclusion Criteria

The inclusion criteria were as follows: articles conducted on humans, those published between 2002 and 2022, articles written in English, articles focusing on the effects of anticoagulants on dementia in AF patients, articles that were peer-reviewed, and full-text articles including subscription articles, as well as meta-analyses, cohort studies, and case-control and observational studies.

Exclusion Criteria

**Figure 1:**
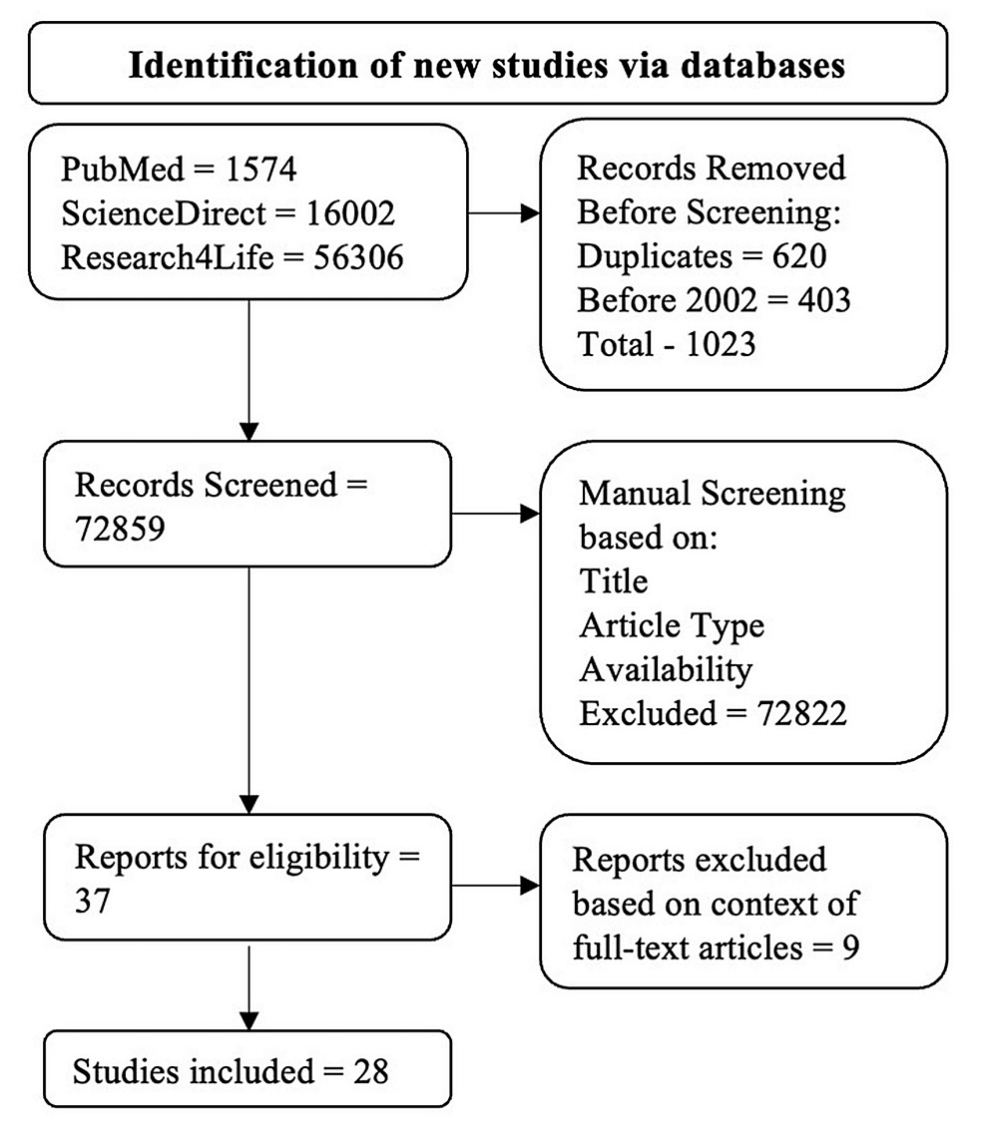
Algorithm used to filter articles based on the study's inclusion and exclusion criteria

The exclusion criteria were as follows: systematic reviews, review articles, and case reports. All non-full-text articles and duplicates were also excluded. The algorithm for inclusion and exclusion is illustrated in Figure 1

Bias

All studies were assessed for bias. All studies showed a medium risk of bias as all of them were based on national studies or retrospective studies on original drug usage. The Grading of Recommendation, Assessments, Development, and Evaluations (GRADE) tool was used to assess the individual risk of bias. GRADE is a tool that evaluates flaws like indirectness, publication bias, and imprecision.

The screening for this literature review was done as per the Preferred Reporting Items for Systematic Reviews and Meta-Analysis (PRISMA) guidelines [5].

Results

Our search generated a total of 73,882 articles: 1,574 from PubMed, 16,002 from ScienceDirect, and 56,306 from ProQuest. Among the exclusions, 620 were duplicate articles, and 403 were published before 2002. This resulted in 1,023 articles being excluded during the automatic screening process, leaving 72,859 articles for manual screening. Articles were manually screened based on the abstract, title, study type, and availability of the full text, resulting in 37 articles being checked for eligibility. Ultimately, 29 articles were chosen for the final analysis.

Anticoagulants assessed in this article were as follows: general OACs, DOACs, general VKAs, and warfarin-specific VKAs. OACs and DOACs were mainly found to reduce the risk of dementia in patients suffering from AF. The effects of VKAs were split between leading to an increase in dementia risk or resulting in some prevention of dementia. Additionally, the studies factored in outpatients who had experienced transient ischemic attacks (TIAs) or strokes to determine the actual effects of anticoagulants on dementia.

Discussion

An average of 4% of patients with AF develop dementia [6,7]. Patients with AF who are not prescribed OACs are more likely to have dementia [8]. This paper looks at OACs (in general), VKAs (in general), and warfarin and DOACs. Also included in this review are a couple of articles assessing antiplatelets' effects on dementia in AF patients with a focus on those with comorbidities. Although antiplatelets are not routinely used in patients with AF only, the subset of patients utilizing them is also included to expand the discussion regarding the treatment of AF in all patients.

OACs

The use of OACs is protective against dementia/cognitive impairment. It contributes to reduced risk as the incidence of dementia per 1000 individuals in OAC groups has been reported to be 2.76 while it is 4.00 in patients not on OACs [7,9-15]. This reduced risk was statistically significant. Zeng et al. observed that the reduction of cognitive impairment/dementia risk with OACs was significant even after adjusting for TIA or stroke [13]. OAC use is associated with an average of 28.8% decrease in the risk of progression to dementia [13,16,17]. Dementia in patients taking OAC had an incidence rate of 6.5 per 1000 person-years, whereas, in non-users, it was 5.8 per 1000 person-years [7]. Dementia was associated with patients with AF who received inadequate OAC treatment (p<0.001) [8]. In agreement with this observation, Jacobs et al. found that the total incidence of dementia increased in each category as the time spent in the supratherapeutic range increased [6]. Patients with the highest percentage of time spent in the supratherapeutic range of INR values were 2.65 times more likely to be diagnosed with dementia compared to patients who spent the lowest time within the supratherapeutic range [6]. Increasing the time in the therapeutic range is associated with decreased risk of dementia [10].

DOACs

In one study, when comparing the DOAC group to the non-DOAC group, the occurrence of dementia per 1000 person-years was 3.14 and 3.69, respectively [15]. Another study reported that patients taking DOACs had a 26% decrease in the rate of dementia [18]. The commonly used DOACs are dabigatran, rivaroxaban, and apixaban, and Chen et al. observed no difference in dementia prevalence between them, with all of them reducing the risk of developing dementia [19]. Choice in DOAC should be based on safety, efficacy, and patient preferences [19]. Concerning OAC and DOAC, Mongkhon et al. observed no significant differences in the risk of reduction of dementia between the two (p=0.588) [17].

VKAs

There have been conflicting findings in studies as to whether VKAs decreased or increased the risk of dementia in AF patients. One study reported that VKA use is associated with a 23% reduced risk of dementia as compared to OAC use (p<0.001) [10,14]. Two studies found a significantly decreased risk of dementia in OAC users compared to those who used VKAs [18,20]. However, two other studies found that those taking VKAs had an increased risk of cognitive impairment compared to those not using VKAs [21,22]. Brangier et al. found that VKA use leads to a continuous decrease in Frontal Assessment Battery scores (p=0.01) [22]. Cognitive impairment was independently associated with VKA use (p=0.028) [22]. Fluindione, a vitamin K antagonist, specifically caused an increased risk of cognitive impairment [22]. Good control of INR with VKA patients was associated with a 27% reduction in dementia diagnosis compared to poor control [18].

Warfarin

Two studies have found that warfarin use does not decrease new-onset dementia incidence [7,23], while several other studies have reported that dementia prevalence is less common in AF patients using warfarin (p<0.023) [17,24-26]. Madhavan et al. observed that, after accounting for confounders, warfarin treatment led to a 22% reduction in the risk of developing dementia [25]. Reduced prevalence of dementia showed a trend toward independent association with the use of warfarin when an exploratory logistic regression was done (p=0.08) [24].

Wong et al. observed that in elderly patients with AF, warfarin use was associated with a lower risk of new-onset dementia when compared to aspirin or no anticoagulation [26]. Studies may show conflicting results because the warfarin given is not as effective due to the difficulty to manage INR levels [7]. Reduced risk of dementia was shown to be correlated to an increase in the time in the therapeutic range of warfarin therapy as determined by an INR between 2 and 3 [25]. Patients on warfarin with >65% of the time in the therapeutic range showed a non-significant trend of less dementia risk than those on warfarin with <65% time in the therapeutic range [26].

VKAs vs. OACs

Patients taking OACs showed a significantly decreased risk of cognitive impairment/dementia compared to those on VKAs [20]. OACs in particular showed a significantly reduced prevalence of dementia when compared to warfarin [9,16,27,28]. Patients taking OACs instead of warfarin were associated with a reduced risk of dementia by up to 22% [28]. However, one study contradicted these results, as it found no clinically significant difference between OAC and warfarin therapy in terms of dementia rates in AF between the ages of 60-79 years [29]. It was demonstrated that OACs were significantly associated with higher dementia rates compared to warfarin use in patients older than 80 years of age [29].

Cadogan et al. observed that dementia prevalence was only slightly lower in patients using DOACs compared to those using VKAs, whereas Bezabhe et al. and Lee et al. found this decrease to be significant [7,18,30]. One study observed that the prevalence of dementia was lower in dabigatran, rivaroxaban, and apixaban users when compared to warfarin users [19,28], while another study observed that edoxaban (DOAC) led to the greatest decrease in the risk of developing dementia [30]. Mongkhon et al. observed no significant difference in the risk reduction of dementia when they compared the use of DOACs vs. warfarin (p=0.373) [17]. Contrary to all studies, Caramelli et al. found a significant difference in the Montreal Cognitive Assessment, suggesting a lower cognitive decline in warfarin users compared to dabigatran, but this finding should be interpreted cautiously [31].

Antiplatelets

The use of antiplatelets for AF is associated with an increased risk of dementia (HR: 1.84) [32]. When comparing the patients on aspirin and clopidogrel (those with comorbidities such as coronary or peripheral artery disease, prior stroke or TIA, prior stent placement, etc.) with patients taking warfarin for the management of AF, those taking aspirin and clopidogrel were found to have an increased risk of developing dementia (p=0.12) [6]. Dual therapy of an antiplatelet agent with OACs was associated with a higher risk of dementia compared to no treatment (p=0.006) [17]. In elderly patients with AF, there was no significant difference in terms of the protective effects of warfarin compared to aspirin for cognitive decline [23]. No apparent reason has been found to explain this observation, but antiplatelets should be used with caution. A summary of the discussion/reviewed findings is presented in Table 1.

**Table 1:** Summary of discussion findings VKA: vitamin K antagonists; OR: odds ratio; HR: hazard ratio; OAC: oral anticoagulant; AF: atrial fibrillation; TICSm/IQCODE: modified Telephone Interview for Cognitive Status/Informant Questionnaire on Cognitive Decline in the Elderly; TAT: thrombin-antithrombin; RR: risk ratio; DOAC: direct-acting oral anticoagulant; MMSE: Mini-Mental State Examination; MPR: medication possession ratio; NTB: neuropsychological test battery; CGNT: computer-generated tests; NOAC: non-vitamin-K-antagonist oral anticoagulants; ARB: angiotensin receptor blocker; TIA: transient ischemic attack

	Author	Country	Design and study population	Findings	Conclusion
1	Annweiler et al., 2015 [21]	France	Cohort study (n=267)	Patients taking VKAs, specifically fluindione, had a 15% higher risk of cognitive impairment. Cognitive impairment was independently associated with VKA use (OR: 17.4, p=0.028)	In geriatric patients, cognitive impairment was more frequently associated with using VKAs, specifically fluindione
2	Madhavan et al., 2018 [25]	United States	Cohort study (n=2,800)	Patients taking warfarin were associated with a decreased risk of developing dementia (HR: 0.80, 95% CI: 0.64–0.99), specifically those within the top two quartiles of the time in the therapeutic range. The time within the therapeutic range of warfarin was correlated to the reduced risk of developing dementia	Patients taking warfarin for a diagnosis of AF experienced a 20% decrease in the risk of developing dementia. As determined by INR, the reduction of time spent in both subtherapeutic and supratherapeutic ranges also showed a reduction in the chance of developing dementia
3	Zhang et al., 2018 [20]	China	Meta-analysis (n=97,595)	When comparing 55,337 patients with AF who took NOACs with 42,258 patients with AF who took VKAs or acetylsalicylic acid, there was a decreased risk of cognitive impairment in the group of patients taking NOACs (HR: 0.80, 95% CI: 0.63–0.98 for fixed-effects model; HR: 0.77, 95% CI: 0.53–1.01 for random-effects model)	Using NOACs may decrease the risk of cognitive impairment in patients with AF compared to acetylsalicylic acid or VKAs
4	Barber et al., 2004 [24]	UK	Cohort study (n=218)	Of 218 patients, 66% were prescribed warfarin, and 22% met the criteria for dementia via the TICSm/IQCODE. F1 + 2, D-dimer, and TAT concentrations were higher in AF patients with dementia (p=0.006, p=0.008, and p=0.003, respectively). There was a trend towards warfarin use being associated independently with reduced occurrence of dementia (OR: 0.52, p=0.08)	Long-term use of warfarin may be protective against dementia development in patients with AF
5	Lin et al., 2021 [14]	China	Meta-analysis (n=613,920)	Comparing patients taking OAC therapy to those receiving no therapy yielded a decrease in the risk of developing dementia [RR (95% CI): 0.72 (0.60–0.86), I^2^=97.2%, p=0.00]	OAC therapy has protective properties in patients diagnosed with AF, and OAC therapy reduces their risk of developing dementia. Long-term future studies should be done with consistent follow-up, especially in younger patients
6	Chen et al., 2018 [19]	United States	Retrospective study (n=307,099)	Patients taking DOACs had lower rates of dementia compared to those taking warfarin (dabigatran HR: 0.85; rivaroxaban HR: 0.85; apixaban HR: 0.80). There were no differences in dementia prevalence between the DOACs (HR: 0.92–1.02)	DOAC users had a lower rate of dementia compared to warfarin users, with no significant difference between DOACs
7	Bezabhe et al., 2022 [7]	Australia	Retrospective study (n=18,813)	Out of 18,813 patients, 425 patients were documented with dementia. The prevalence of dementia was significantly lower in patients using OACs (p<0.001). For patients using DOACs, dementia incidence was significantly lower than for those using warfarin (p=0.002) and those not using OACs (p<0.001). There was no significant difference in dementia between non-OAC users and warfarin users (p=0.723)	DOAC use may result in a lower incidence of dementia in AF patients compared to warfarin or no OACs
8	Brangier et al., 2018 [22]	France	Cohort study (n=378)	VKAs were associated with worse/low Frontal Assessment Battery scores at baseline (p=0.026) and decreased after 24 months (p=0.01). VKA use was not associated with changes in MMSE score at any time	VKA use was associated with a decreased Frontal Assessment Battery and executive dysfunction with no change in MMSE score compared to counterparts
9	Lee et al., 2021 [30]	Korea	Retrospective cohort study (n=72,846)	The use of DOAC (edoxaban), compared with warfarin, decreased the risk of dementia in the Asian population (HR: 0.830, 95% CI: 0.740–0.931)	In patients with diagnosed AF, DOAC showed comparable risk reduction to warfarin. DOACs, especially edoxaban, had a beneficial effect on patients within the age group of 65-74 years and a history of stroke in the decrease of the development of dementia
10	Mongkhon et al., 2019 [10]	Thailand	Meta-analysis (6 studies)	OAC use has a protective effect that reduces the risk of dementia in patients with AF (RR: 0.79, 95% CI: 0.67–0.93, I^2^=59.7%, p=0.005)	The use of OACs is protective against dementia and contributes to reduced risk
11	Komatsu et al., 2021 [15]	Japan	Retrospective cohort study (n=17,962)	Patients taking OAC with an MPR of above 90% were associated with a significantly lowered risk of developing dementia (HR: 0.45, 95% CI: 0.25–0.81, p=0.008). There was a significant difference in developing dementia when comparing groups with an MPR above 90% to non-OAC groups. There was a substantial decrease in the development of dementia in the group taking OAC with an MPR above 90% (log-rank test: p=0.006)	In elderly patients diagnosed with AF with an MPR of above 90% and taking OAC, there was a decrease in the risk of developing dementia. OAC in elderly patients serves as a protective effect against developing dementia
12	Mongkhon et al., 2020 [17]	Thailand	Retrospective cohort study (n=84,521)	OAC use is associated with a reduced risk of dementia compared to no OAC use (HR: 0.90, 95% CI: 0.85–0.95; p<0.0001)	The use of OACs is protective against dementia and contributes to reduced risk
13	Cadogan et al., 2021 [18]	UK	Retrospective study (n=39,200)	DOAC use for AF was associated with a 16% reduction in dementia compared with VKA and a 26% reduction in the incidence of mild cognitive impairment	DOACs showed a reduced risk of dementia and mild cognitive impairment compared to VKA users
14	Caramelli et al., 2022 [31]	Brazil	Randomized study (n=5,523)	MMSE, NTB, and CGNT scores in the dabigatran minus group suggest a less cognitive decline in warfarin (p=0.75, p=0.06, p=0.02, respectively)	There was a lower cognitive decline in patients assigned warfarin
15	Kim et al., 2021 [28]	Korea	Cohort study (n=53,236)	NOACs like dabigatran, rivaroxaban, and apixaban were linked to a lower risk of developing dementia (HR: 0.78, 95% CI: 0.69–0.90). When comparing rivaroxaban to dabigatran, rivaroxaban showed a decreased risk of developing dementia (HR: 0.83, 95% CI: 0.74–0.92)	With NOAC therapy in patients diagnosed with non-valvular AF, NOACs were associated with a lower risk of developing dementia than warfarin therapy
16	Kim et al., 2019 [12]	Korea	Cohort study (n=262,611)	Patients taking OAC therapy for a diagnosis of AF experienced a preventative effect from developing dementia, whereas those with a higher CHA_2_-DS_2_-VASc score had a higher risk of developing dementia. (HR: 0.61, 95% CI: 0.54–0.68)	The use of OAC therapy in patients was linked to decreased risk of developing dementia
17	Cheng et al., 2018 [9]	China	Meta-analysis (n=471,057)	OACs significantly reduced the prevalence of dementia in patients with AF (p<0.00001). Compared to warfarin, NOACs significantly reduced the prevalence of dementia (p<0.00001)	OACs and NOACs reduced the prevalence of dementia in patients with AF
18	Jacobs et al., 2015 [6]	United States	Cohort study (n=992)	In patients taking warfarin therapy for AF, those whose INR was above a value of 3 were linked to a higher incidence of developing dementia in more than a ¼ of participants and a 2.4 times greater risk (p=0.04)	Patients taking anticoagulant therapy who experience too much time in an over-coagulable state are at a higher risk of developing dementia. This predisposes the patient to microbleeds, which could be the mechanism causing the incidence of dementia to increase
19	Mavaddat et al., 2014 [23]	UK	RCT (n=973)	No evidence was found to suggest that anticoagulants provide clinically significant protection against cognitive decline in patients with AF aside from preventing stroke	In elderly patients with AF, no significant difference was seen in the protective effects of warfarin compared to aspirin for cognitive decline
20	Hsu et al., 2021 [27]	Taiwan	Cohort study (n=25,089)	When comparing patient groups of warfarin to NOAC, NOAC use was linked to a lower risk of developing dementia, particularly in patients aged 65-75 years with a high risk of bleeding and stroke	The patients taking NOACs instead of warfarin for AF had a lower associated risk and incidence of dementia than patients taking warfarin therapy for the same condition
21	Søgaard et al., 2019 [29]	Denmark	Cohort study (n=33,617)	Use of NOACs was significantly associated with higher dementia rates compared to warfarin use in patients older than 80 years (2.16 events/100 person-years vs. 1.70 events/100 person-years; weighted HR: 1.31, 95% CI: 1.07–1.59)	No clinically significant difference was seen in the use of NOAC vs. warfarin in dementia rates in patients with AF
22	Ding et al., 2018 [32]	Sweden	Cohort study (n=2,685)	Of the 2,685 patients, 243 had AF; at nine years, 522 patients had AF and 399 patients developed dementia. AF was associated with vascular and mixed dementia. Those with AF taking anticoagulants were associated with a 60% decreased risk for dementia	AF is associated with cognitive decline, and anticoagulant use may reduce the risk of dementia
23	Friberg et al., 2019 [11]	Sweden	Retrospective cohort study (n=456,960)	Patients in Sweden diagnosed with AF were given OACs, which were associated with a lower incidence of dementia [subhazard ratio (sHR): 0.62, 95% CI: 0.48–0.81]. The decrease was 12% and mainly benefitted individuals over 65 years of age (sHR: 0.88, 95% CI: 0.72–1.00)	Patients diagnosed with AF who are at low risk and take OAC have a lower risk of developing dementia. Patients above the age of 65 years benefit from the decrease in the chance of developing dementia regardless of their stroke risk score
24	Friberg et al., 2018 [16]	Sweden	Retrospective cohort study (n=444,106)	Patients treated with anticoagulant therapies for a diagnosis of AF benefitted from a 29% decrease in the risk of developing dementia compared to patients not taking OAC (HR: 0.71, 95% CI: 0.68–0.74)	Patients with AF have a higher risk of developing dementia when not taking any OAC therapy. This shows the association of a protective effect of administering OAC in patients with AF to reduce their risk of developing dementia
25	Viscogliosi et al., 2017 [8]	Italy	Cross-sectional retrospective study (n=316)	Patients with AF who received inadequate OAC were associated with dementia independent of age (OR: 1.33, 95% CI: 1.11–1.46, p<0.001)	Underuse of anticoagulants in older patients with AF is associated with dementia
26	Wändell et al., 2019 [33]	Sweden	Observational study (n=160,251)	The use of ARBs was associated with a lower risk of dementia in patients with AF (HR: 0.87, 99% CI: 0.78–0.98). Loop diuretics were associated with a greater risk for dementia among patients with AF aged 65–84 years (HR: 1.16, 99% CI: 1.00–1.35)	ARBs are associated with decreased risk of dementia, and loop diuretics are associated with a higher risk of dementia
27	Zeng et al., 2019 [13]	China	Meta-analysis (n=454,273)	The use of anticoagulants in patients with AF was associated with decreased risk for cognitive impairment as compared with non-anticoagulation (RR: 0.72, 95% CI: 0.69–0.75, I^2^=11.5%)	The use of anticoagulants in patients with AF was associated with cognitive benefits independent of TIA or stroke
28	Wong et al., 2022 [26]	China	Observational study (n=3,284)	In patients with AF, warfarin was associated with significantly less risk of dementia (HR: 0.14%, 95% CI: 0.05–0.36, p<0.001)	In elderly patients with AF, warfarin use was associated with a lower risk of new-onset dementia than aspirin or no anticoagulation therapy

This study has a few limitations. Primarily, most of the articles selected were retrospective studies. While clinical trials may not be ethical to an extent, they provide a real-time analysis of how the interplay of anticoagulants and dementia would work. Also, we excluded animal studies as the effects on humans were the primary concern, and including them would have elucidated a possible mechanism for why the phenomenon we address occurs.

## Conclusions

Overall, the direct link between anticoagulant use and the incidence of dementia is not fully understood, and this paper attempts to bridge the gap between the two. Most studies supported this association, especially when comparing OACs, DOACs, VKAs/warfarin, and antiplatelet therapy in patients diagnosed with AF. When analyzing the use of OACs, most studies suggested that OAC therapy decreased the risk of developing dementia. When comparing patients taking DOACs like dabigatran and rivaroxaban to patients taking no OACs, there was also a decrease in the occurrence of dementia. VKA usage yields conflicting results when assessing the risk reduction of dementia, with some studies indicating VKAs increase dementia risk while others suggest the opposite. Warfarin therapy specifically was associated with a decrease in the incidence of dementia. With all anticoagulants, it is essential to monitor the INR as research has shown that maintaining an optimal range is associated with a decreased risk of dementia. Lastly, patients taking antiplatelet drug therapy such as aspirin and clopidogrel were linked to an increased risk of dementia compared to those taking warfarin therapy. The link between OAC use and the risk of developing dementia is a common theme in many studies. This warrants further exploration of the connection between the two as it might lead to a better understanding of the mechanism of its protective effects and help prevent the onset of cognitive impairment.
